# Normalization of eosinophil count is predictive of oxygen weaning over the course of COVID-19 infection among hospitalized adults during the first wave of 2020 pandemic

**DOI:** 10.3389/fimmu.2024.1381059

**Published:** 2024-05-24

**Authors:** Benjamin Davido, Karim Jaffal, Azzam Saleh-Mghir, Isabelle Vaugier, Stephane Bourlet, Pierre De Truchis, Djillali Annane

**Affiliations:** ^1^ Maladies Infectieuses, Université Paris-Saclay, Assistance Publique des Hôpitaux de Paris (AP-HP) Hôpital Raymond Poincaré, Garches, France; ^2^ Centre d’Investigation Clinique (Inserm CIC 1429), Université Paris-Saclay, Assistance Publique des Hôpitaux de Paris (AP-HP) Hôpital Raymond Poincaré, Garches, France; ^3^ Médecine Intensive Réanimation, Université Paris-Saclay, Assistance Publique des Hôpitaux de Paris (AP-HP) Hôpital Raymond Poincaré, Garches, France

**Keywords:** eosinophil count, COVID-19, normalization, oxygen, eosinopenia, oxygen weaning, Covid

## Abstract

**Background:**

Understanding COVID-19 outcomes remains a challenge. While numerous biomarkers have been proposed for severity at admission, limited exploration exists for markers during the infection course, especially for the requirement of oxygen therapy. This study investigates the potential of eosinophil count normalization as a predictor for oxygen weaning during the initial wave of the pandemic.

**Methods:**

A retrospective study was conducted between March and April 2020 (first wave) among adults admitted directly to a medicine ward. Biological abnormalities, including lymphocyte count, eosinophil count, and C-reactive protein (CRP), were gathered daily during the first week of admission according to oxygen level. In case of worsening, oxygen level was censored at 15 L/min. The primary aim was to assess whether eosinophil count normalization predicts a subsequent decrease in oxygen requirements.

**Results:**

Overall, 132 patients were admitted, with a mean age of 59.0 ± 16.3 years. Of the patients, 72% required oxygen, and 20.5% were admitted to the intensive care unit after a median delay of 48 hours. The median CRP at admission was 79 (26–130) mg/L, whereas the eosinophil count was 10 (0–60)/mm^3^. Eosinophil count normalization (≥100/mm^3^) by day 2 correlated significantly with decreased oxygen needs (<2 L) with hazard ratio (HR) = 3.7 [1.1–12.9] (p = 0.04). Likewise, CRP < 80 mg/L was associated with reduced oxygen requirements (p < 0.001). Predictors, including underlying chronic respiratory disease, exhibited a trend toward a negative association (p = 0.06).

**Conclusion:**

The study highlights the relationship between eosinophil count and CRP, with implications for predicting oxygen weaning during COVID-19. Further research is warranted to explore the relevance of these biomarkers in other respiratory infections.

## 1 Introduction

After 4 years of the pandemic, COVID-19 is a well-known disease, particularly regarding the physiopathology of its pneumonia related to a severe cytokine storm (1), whose prognosis has been associated with the use of corticosteroids (2). In such clinical presentations, the initial need for oxygen therapy has been correlated with the severity of lung involvement, typically supported by extended ground-glass opacities observed on CT scans (3). It should also be emphasized that the emergence of the Omicron variant, in comparison to the Delta variant, was a total game-changer concerning clinical outcomes but was also associated with a reduction in chest CT severity (4, 5).

Even if the management of hospitalized patients with COVID-19 largely relies on CT imaging, comorbidities, and vaccination status (6), predicting patient outcome remains challenging. Better anticipating a patient's respiratory worsening allows for more tailored medical treatment. Indeed, possible therapeutics are currently dependent on oxygen supply and the promptness of consultation to consider antiviral drugs (7) or, conversely, corticosteroids.

Moreover, biological abnormalities especially biomarkers such as C-reactive protein (CRP) and procalcitonin (PCT) or neutrophil and lymphocyte count are deemed to be factors of prognosis at admission during COVID-19 (8–10), as well as other biomarkers such as interleukin-6, lactic dehydrogenase, or D-dimer (11). Even eosinophil count has been proposed to distinguish COVID-19 from other causes upon admission (12). Regarding another aspect, eosinophil count is a forgotten biomarker that has shown interest in the intensive care unit (ICU) to predict patient outcomes during the first 7 days of admission (13). Similarly, the resolution of eosinopenia (low eosinophil count) has been proposed as a useful parameter to monitor in predicting the response to therapy in patients hospitalized in a medicine ward for infection (14). Additionally, a recent study by Partouche et al. (15) has suggested that the normalization of eosinophil count (≥100/mm^3^) after 48 hours of appropriate regimen was related to a ninefold increase in the likelihood of favorable outcome among elderly infected patients. To the best of our knowledge, there is no biomarker to predict oxygen dependence during ongoing COVID-19 infection.

Our work intends to assess the kinetics of eosinophil count over time based on oxygen levels during the acute phase of this viral infection, namely, COVID-19, during this first wave of the epidemic when no specific drugs were approved. Moreover, at that time, COVID-19 served as an excellent model for investigation, particularly considering that patients underwent routine daily biological follow-ups.

## 2 Methods

### 2.1 Setting

This was a single-center and retrospective study during the first wave of COVID-19 (from early March to late April 2020) of adults admitted to a medicine ward for a COVID-19 infection confirmed by SARS-CoV-2 RT-PCR in a tertiary care hospital, Hôpital Raymond Poincaré (AP-HP), Garches, France.

### 2.2 Data collection

The following data were collected from the patients’ medical charts:

− Patient characteristics: age, sex, diabetes, cardiovascular risk factors, smoking habits, obesity, chronic pulmonary disease, and Charlson comorbidity index (CCI) (16).
− Infection and treatment characteristics: delay between onset of symptoms and admission, percentage of lung injuries on CT scan if applicable and available, requirement of ICU support with invasive ventilation, and associated therapeutic strategies (e.g., oxygen and associated drugs). Of note at the time of the study in our hospital [before the RECOVERY protocol (2)], patients were not eligible for corticosteroids in the medicine ward and were proposed to be treated with steroids in randomized clinical trials only in the ICU.
− Biological abnormalities, including polymorphonuclear neutrophils (PMNs), lymphocyte count, eosinophil count, and CRP were gathered daily during the first week of admission according to oxygen level, starting from the date of admission. In case of worsening requiring ICU, the oxygen level was censored at 15 L/min.

### 2.3 Objective

The aim of the study was to investigate whether normalization of eosinophil count could predict a decrease in oxygen requirements and potentially delay treatment initiation.

### 2.4 Statistical analysis

Values were presented as median with 95% CI on figures or interquartile range (IQR) in the main text, and statistical analysis utilized the chi-square square test. Multivariate analyses used linear logistic regression. Statistical significance was set at 0.05 (two-tailed test). All statistical calculations were performed using Prism GraphPad software version 9.1.1.

### 2.5 Compliance with ethical standards

All procedures performed in studies involving human participants were in accordance with the ethical standards and with the 1964 Declaration of Helsinki and its later amendments or comparable ethical standards. This study has passed the CESREES/Health Data Hub regarding ethics committee approval (MR1811190620) and is registered on ClinicalTrials.gov (NCT04453501). As part of an anonymous and retrospective study, a non-opposition and information letter was sent to participants afterward.

## 3 Results

### 3.1 Description of the population at baseline

Between March 5 and April 25, 2020, 132 patients with COVID-19 were admitted. Baseline characteristics are summarized in **Table 1**. The mean (± SD) onset of symptoms was 7 ± 4 days. Of the patients, 72% required oxygen supply, with a median (IQR) value of 2.25 (2–4) L/min. Of note, ultimately, an additional 10 patients required oxygen during admission. Among individuals who underwent a CT scan (n = 103), 53.5% had moderate (25%–50%) to severe (>50%) lung involvement. Twenty-seven patients (20.5%) required ICU due to worsening condition. Among them, 18 patients received corticosteroids after a median delay of 2 (1–8) days from admission.

**Table 1:** Baseline characteristics of patients.

Characteristics at baseline	N = 132
Age (year), mean ± SD	59.0 ± 16.3
Sex (M), no. (%)	86 (65)
Obesity, no. (%)	14 (10.6)
Diabetes, no. (%) Chronic kidney failure, no. (%) Chronic respiratory disease, no. (%) Chronic cardiac disease, no. (%) Immunocompromised, no. (%)	25 (18.9) 4 (3) 22 (16.7) 57 (43.2) 11 (8.3)
CCI*, no. (%)	
0	24 (18)
1–2	47 (36)
3–4	31 (23)
≥5	30 (23)
Pulmonary CT scan, no. (%)	103 (78)
Normal	7 (7)
Limited injury (<10%)	11 (11)
Mild injury (10%–25%)	30 (29)
Moderate injury (25%–50%)	41 (40)
Severe injury (>50%)	14 (14)
Lymphocyte count <1,000/mm^3^, no. (%)	60 (45.5)
PMN count >8,000/mm^3^	12 (9.1)
CRP mg/L, mean ± SD Ferritin µg/L, mean ± SD	84.59 ± 70.31 1,070 ± 757
Oxygen (yes), no. (%)	94 (72)
≤2 L/min	47 (36)
2–5 L/min	37 (28)
≥6 L/min	10 (8)
Treatment strategies, no. (%)	
No effective drugs	114 (86.4)
Corticosteroids in ICU	18 (13.6)

Obesity defined by a body mass index ≥30 kg/m^2^. N, number; %, percent; SD, standard deviation; M, men; *CCI, Charlson Comorbidity Index; CRP, C-reactive protein; PMN, polymorphonuclear leukocyte; CT, computed tomography.

### 3.2 Biological abnormalities

At admission, the following median (IQR) values were revealed: CRP = 79 (26–130) mg/L, lymphocyte count = 1,005 (730–1,380)/mm^3^, and eosinophil count = 10 (0–60)/mm^3^.

**Figure 1:**
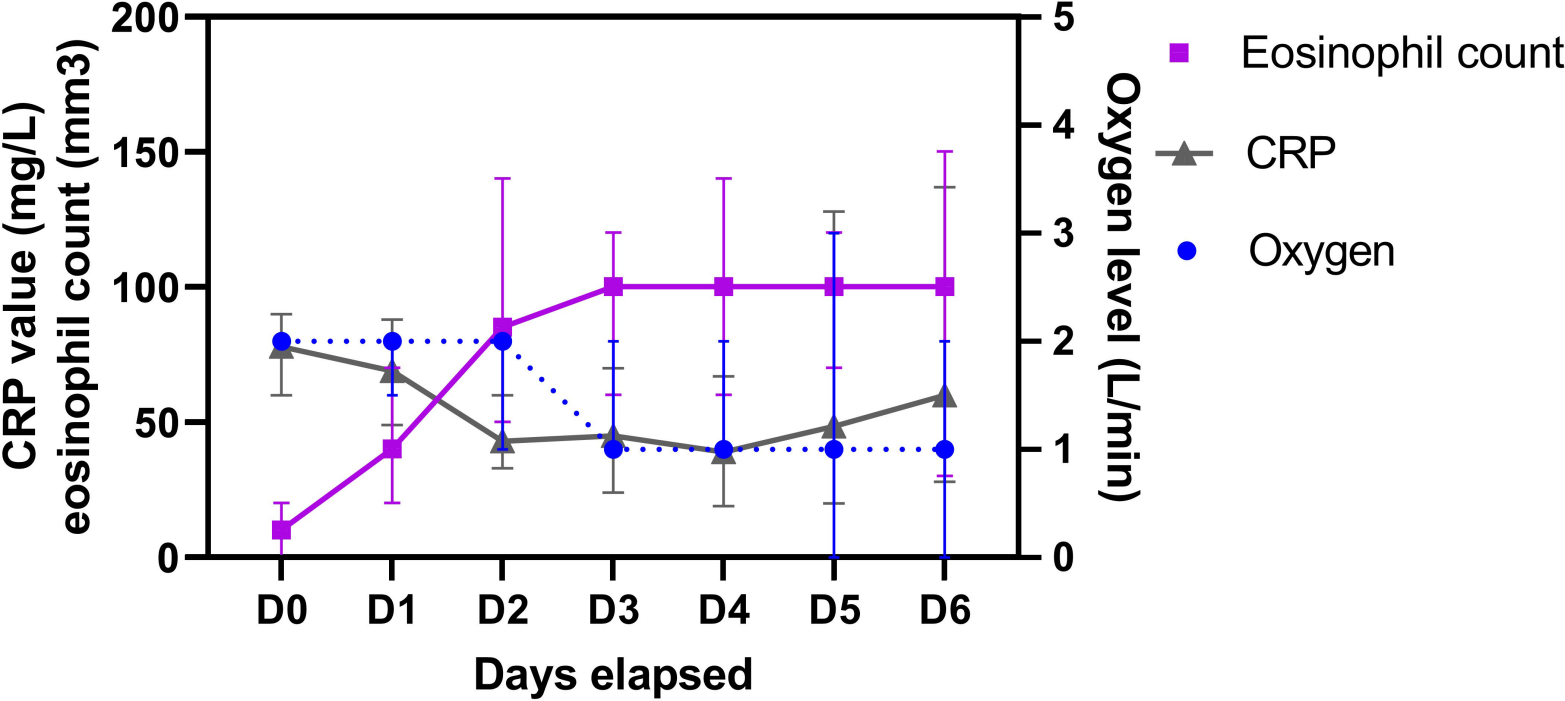
Behavior of eosinophil count over time from the admission (D0) according to C-reactive protein (CRP) value and oxygen level requirements. Values represent median with 95% confidence interval (CI).

Eosinophil count increased continuously until normalization (median ≥100/mm^3^) from day (D) 0 to D3, while the oxygen level started to decrease on day 3 without any rebound observed thereafter (**Figure 1**). Similarly, median CRP decreased to 43 mg/L on day 2 but ultimately increased again. Of note, patients from this subgroup, whose CRP levels eventually increased again, were primarily admitted to the ICU (n = 9/13) and were at a higher risk of mortality (38% vs. 5%; p = 0.002).

Patients requiring <2 L/min of oxygen were significantly associated with an eosinophil count (≥100/mm^3^) on day 2 (70.0% vs. 31.5%), with OR = 5.5 [2.19–13.8] (p < 0.001) in univariate analysis (see **Table 2**).

**Table 2:** Potential factors associated with oxygen need <2 L/min after admission.

Variables	n/N	Univariate model		Multivariate model	
		HR [95% CI]	p-Value	HR [95% CI]	p-Value
Characteristics at baseline					
Age ≥ 60 (years)	65/132	0.45 [0.21–0.96]	0.04	1.91 [0.49–7.4]	0.35
Sex (M)	86/132	0.8 [0.37–1.8]	0.59	–	–
Obesity (yes)	14/132	0.55 [0.15–1.99]	0.36	–	–
Diabetes, no. (%)	25/132	0.94 [0.37–2.35]	0.89	–	–
Chronic respiratory disease, no. (%)	22/132	0.31 [0.10–0.9]	0.03	0.16 [0.02–1.08]	0.06
Chronic cardiac disease, no. (%)	57/132	0.79 [0.38–1.7]	0.55	–	–
Immunocompromised, no. (%)	11/132	0.56 [0.15–2.1]	0.37	–	–
Biological abnormalities at D2					
PMN count <8,000/mm^3^	82/94	2.37 [0.43–12.9]	0.31	–	–
Lymphocyte count ≥1,000/mm^3^	49/94	3.47 [1.39–8.7]	0.008	0.49 [0.11–2.2]	0.35
Eosinophil count ≥100/mm^3^	45/94	5.5 [2.19–13.8]	<0.001	3.71 [1.06–12.9]	0.04
CRP <80 mg/L	56/87	22.1 [4.71–103]	<0.001	26.28 [4.07–169.8]	<0.001
Treatment strategies					
No treatment**	114/132	1*	–	1*	**-**
Corticosteroids	18/132	0.04 [0.05–0.3]	0.003	0.12 [0.08–1.7]	0.11

1* indicates the reference category. No treatment** was defined as patients who have had no treatment including lopinavir–ritonavir or antibiotics. Multivariate Cox model regression was used to identify the potential factors associated with oxygen need <2 L/min at day 2 of admission.

n/N, number/total; HR, hazard ratio; CI, confidence interval; PMN, polymorphonuclear; CRP, C-reactive protein.

"-" Not applicable.

**Figure 2:**
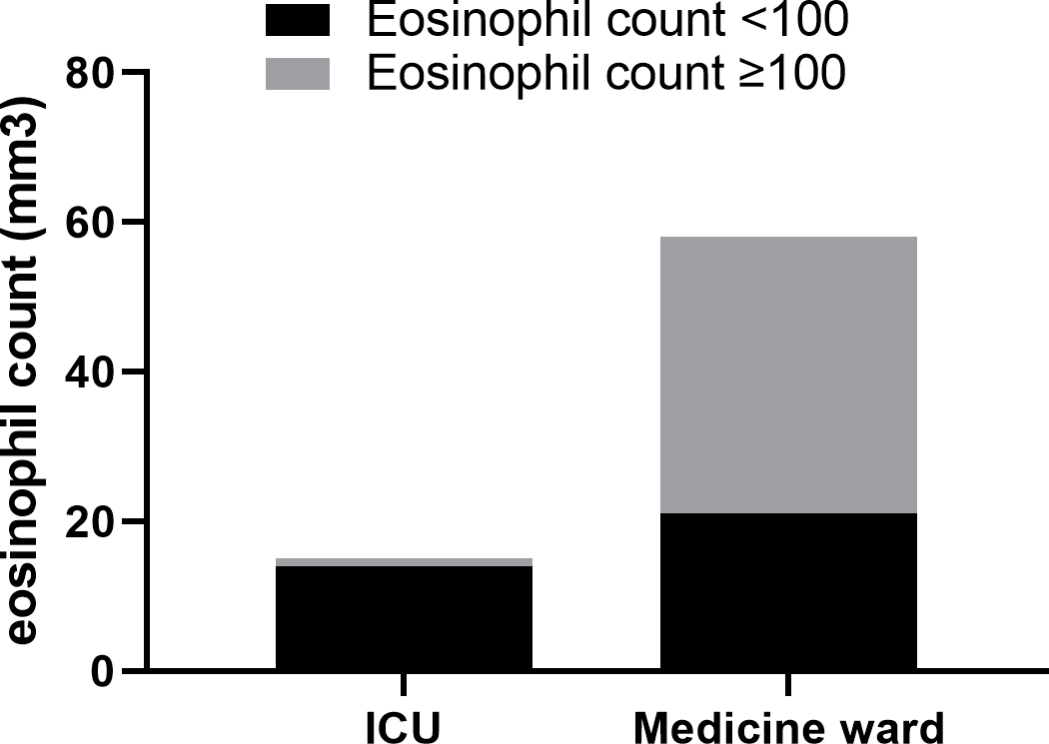
Distribution of patients between intensive care unit (ICU) and medicine ward, according to eosinophil count (<100/mm^3^ or ≥100/mm^3^).

At D2, eosinophil count <100/mm^3^ was significantly more prevalent in patients who were transferred to the ICU (88%) than hospitalized in the medicine ward (39%), with an OR = 11.4 [3.1–38] (p < 0.0001) (see **Figure 2**). Likewise, in univariate analyses, lymphocyte count and CRP values were potential factors associated with oxygen need <2 L/min after admission (p = 0.008 and <0.001, respectively).

Moreover, considering patients who underwent a pulmonary CT scan and had eosinophil count measured until late follow-up (n = 58), lung injury extension (≤25% or >25%) was not predictive of eosinophil count normalization (≥100/mm^3^) at D3 (42% vs. 58%; p = 0.27).

The behavior of eosinophil count over time that tends to normalization was not reproducible using either PMN or lymphocyte count during follow-up, in comparison to oxygen level. Indeed, PMNs continuously increased regardless of normalization, whereas lymphocyte count remained stable (**Supplementary Figure 1**).

In multivariate analyses, factors associated with an oxygen level <2 L/min were eosinophil count ≥100/mm^3^ (p = 0.04) and CRP < 80 mg/L (p < 0.001). Conversely, there was a trend toward underlying chronic respiratory disease as a negative association (p = 0.06) (see **Table 2**).

## 4 Discussion

Our study highlights that the behavior of two biological markers (eosinophil count and CRP) is associated with oxygen weaning during ongoing COVID-19 infection. Indeed, normalization of eosinophil count appears after day 3 and is related to decreased oxygen need (<2 L) within 48 hours, as long as CRP remains below 80 mg/L.

Furthermore, a CRP greater than 75 mg/L has been recently described in a systematic review as a laboratory predictor of severe COVID-19 on admission (17), reflecting the high blood inflammation induced by the elevated cytokine responses during the first wave of COVID-19 pneumonia. However, it should be noted that CRP is not specific to COVID-19 and is related to systemic blood inflammation, which varies significantly between variants over the course of the COVID-19 pandemic (18). Moreover, mean CRP values did not achieve normalization after 3 days of admission, contrary to eosinophil count (≥100/mm^3^), which is a more convenient assay for clinical evolution at no extra cost on regular complete blood count.

To the best of our knowledge, our findings regarding eosinophil count normalization as a marker of oxygen weaning have never been described. Indeed, although eosinopenia has been reported as a marker of severity during the course of severe COVID-19 infection (19), in particular during the first and second waves of the pandemic (20), this biological marker has only been proposed as a marker of unfavorable outcome. Recently, Yan et al. (21) demonstrated that eosinophil recovery in patients with <50 eosinophils/µL was associated with significantly reduced ICU admission (adjusted hazard ratio (aHR) = 0.25 [0.11–0.61]) and in-hospital mortality (aHR = 0.44 [0.29–0.65]), but without significant rates in mechanical ventilation. This longitudinal study, conducted during the first and second waves of the pandemic, also suggests that the lack of eosinophil recovery events might be indicative of patients at risk for future progression to severe COVID-19.

When looking deeper into the physiopathology of eosinophil count during infection, it should be remembered that Zappert et al. (22) first described eosinopenia in 1893 as a non-specific biological marker of systemic inflammatory response syndrome, more than infection. Two main hypotheses explain the mechanism of eosinopenia during infection: i) bone marrow consideration for the production of other lineages of White blood cells (WBCs) and ii) the sequestration of eosinophil count at the site of the infection (in the case of COVID-19, the pulmonary tract). It is noteworthy that recently de Vries et al. (23) demonstrated that at the time of admission, a combination of eosinopenia and increased markers of endothelial damage VCAM and endothelin are characteristic of COVID-19, revealing particular interest in eosinophil count during the latter. While some might argue that during COVID-19 pneumonia, eosinophils are trapped in the pulmonary parenchyma, Cauchois et al. (20), supported by histological findings, reported no eosinophil infiltration in the lungs of patients with eosinopenia. These findings support that peripheral blood eosinophils behave according to the magnitude of immune hyperactivation during moderate-to-severe infection, which is responsible for eosinopenia. In our present study, we observed rapid recovery from eosinopenia in cases of favorable outcome, which is clinically assessed by oxygen weaning.

Furthermore, our study shows a close relationship between normalization of eosinophil count and CRP weaning, a phenomenon previously described during the course of COVID-19 infection (20). Although in our study a CRP threshold below 80 mg/L seems more accurate (p < 0.001) than eosinophil count (0.04) to predict oxygen weaning, its confidence interval is wider due to low statistical power. It is noteworthy that previous studies on COVID-19 assessed the level of oxygen support based on various parameters, encompassing absolute neutrophil count, CRP, and diverse ratios involving lymphocyte count, yet none of them incorporated eosinophils (24–26).

For conditions unrelated to COVID-19, historical predictors of oxygen weaning have been related to patients’ characteristics and dyspnea scales in individuals undergoing pulmonary rehabilitation (27). Similarly, a previous study described the outcomes of respiratory function tests associated with non-invasive ventilation weaning in cases of acute exacerbation of chronic pulmonary disease (28). Having a reliable biological marker, such as CRP or eosinophils, readily available to predict oxygen weaning in medicine ward is relevant, considering that a substantial number of inpatients remain admitted after visiting the emergency room, especially during waves of winter seasonal respiratory tract infections.

Moreover, the negative association (though not statistically significant) between the use of corticosteroids in our study and oxygen levels is not surprising, given that corticosteroids were initiated in cases of deterioration in the ICU. Likewise, age over 60 years was not significantly associated with oxygen need, which may seem counterintuitive given that age is a major factor in severe COVID-19. This may be attributed to confounding factors related to ICU admission, with patients more frequently aged 60 years (mean 66 years vs. 57 years; p = 0.05). Our study has several limitations. First, this single-center study was conducted during the initial wave of the pandemic when a substantial number of hospitalized COVID-19 patients exhibited severe morbidity, unlike the current situation shaped by Omicron and its subvariants in 2024. Second, during this phase of the pandemic at our medical institution, corticosteroids were not routinely prescribed prior to ICU admission. It is evident that when dexamethasone is prescribed, monitoring eosinophil count becomes impractical, given that steroids are accountable for eosinopenia. Nevertheless, it should be noted that the WHO guidelines specifically recommend the use of steroids for patients experiencing at least severe COVID-19 infection, characterized by an oxygen saturation level below 90% on room air (29), typically necessitating supplemental oxygen therapy of at least 2 L/min. Consequently, our surveillance of eosinophil count may remain relevant for patients under oxygen monitoring and not receiving steroids. Third, our study involved a limited sample size and did not permit in-depth investigations into confounding factors, which today would include considerations such as vaccination status, antiviral treatments, and the impact of different COVID-19 variants.

It is important to note that neither eosinophil counts nor eosinopenia is in any way specific to COVID-19 as we already argued (30). However, we believe their kinetics over time are of interest, even in cases of viral infection. Further studies evaluating these two biomarkers (CRP and eosinophil count) could be relevant for other respiratory tract infections, such as bacterial pneumonia under an appropriate regimen. Additionally, Davido et al. (31) previously demonstrated that the normalization of eosinophil count over time is associated with appropriate and effective antimicrobial therapy, suggesting a potentially similar impact on oxygen requirements during the course of treatment.

## 5 Conclusion

Our findings revealed interest in a simple biomarker such as normalization of eosinophil count during the course of COVID-19 infection to predict oxygen weaning. Although CRP showed a higher hazard ratio, the requirement for a specific threshold may be a limitation in its application in daily clinical practice, particularly with the emergence of Omicron variants, which demonstrate a lower systemic inflammatory response than during previous waves.

## Data availability statement

The raw data supporting the conclusions of this article will be made available by the authors, without undue reservation.

## Ethics statement

This study has passed the CESREES/Health Data Hub regarding ethics committee approval (MR1811190620) and is registered on ClinicalTrials.gov (NCT04453501). The studies were conducted in accordance with the local legislation and institutional requirements. Written informed consent for participation in this study was provided by the participants’ legal guardians/next of kin. Written informed consent was obtained from the individual(s) for the publication of any potentially identifiable images or data included in this article.

## Author contributions

BD: Conceptualization, Formal analysis, Investigation, Methodology, Software, Writing – original draft. KJ: Validation, Writing – review & editing. AS-M: Supervision, Writing – review & editing. IV: Conceptualization, Formal analysis, Methodology, Writing – original draft. SB: Investigation, Writing – review & editing. PD: Conceptualization, Supervision, Validation, Writing – review & editing. DA: Validation, Visualization, Writing – review & editing.

## COVID-19 RPC Team

Department of Intensive Care

Djillali Annane, MD, PhD (1,2,5) Xavier Ambrosi, MD (4) Suzanne Amthor, MD (1) Rania Bounab, MD (1,2) Ryme Chentouh, MD (1) Bernard Clair, MD (1) Abdallah Fayssoil, MD (1,2,5) Diane Friedman, MD (1) Nicholas Heming, MD, PhD (1,2,5) Virginie Maxime, MD, (1) Pierre Moine, MD, PhD (1,2,5) Myriam Niel Duriez, MD (1) David Orlikowski, MD, PhD (1,2,5,8) Francesca Santi, MD (1,2)

Pharmacy

Frédérique Bouchand, PharmD (1) Muriel Farcy-Afif, PharmD (1) Hugues Michelon, PharmD, MSc (1) Maryvonne Villart, PharmD (1) Research Staff Isabelle Bossard (8) Tiphaine Barbarin Nicolier (1) Stanislas Grassin Delyle, MCUPH (2,3,5) Elodie Lamy (2,5) Camille Roquencourt, MD (5) Gabriel Saffroy (2) Etienne Thevenot (5)

Department of Intensive Care Interns

Baptiste Abbar (1) Steven Bennington (1) Juliah Dray (1) Pierre Gay (1) Elias Kochbati (1) Majistor Luxman (1) Myriam Moucachen (1) Alice Pascault (1) Juan Tamayo (1) Justine Zini (1) 

Department of Anesthesia, Perioperative Care, and Pain

Marie Boutros, MD (1) Anne Lyse Bron, MD (11) Denys Coester, MD (12) Etiennette Defouchecour, MD (11) Brigitte Dosne Blachier, MD (11) Léa Guichard, MD (1) Damien Hamon Pietrin, MD, PhD (1) Hakim Khiter, MD (1) Valéria Martinez, MD, PhD (1,2,6) Simone Meuleye, MD (1) Suzanne Reysz, MD (1) Sebastien Schitter, MD (1) Chawki Trabelsi, MD (1)

Pediatric Critical Care Unit

Helge Amthor, MD, PhD (1,2,7) Jean Bergounioux MD (1,2,5) Maud Guillon, MD (1) Amal Omar, MD (1) Laboratory of Physiology Frédéric Lofaso, MD, PhD (1,2,7,10) Helene Prigent, MD, PhD (1,2,7,10)

Department of Rehabilitation and Physical Medicine

Djamel Bensmail, MD, PhD (1,2,7,10) Pierre Denys, MD, PhD (1,2,7,10) Charles Joussain, MD, PhD (1) Lauren Kagane, MD (1) Thibaut Lansaman, MD (1) Hélène Le Liepvre, MD (1) Antoine Leotard, MD, MS (1) Jonathan Levy, MD, MS (1,2,7,10) Claire Malot, MD (1) Julie Paquereau, MD (1) Celia Rech, MD (1)

Department of Rehabilitation Interns

Florence Angioni (1) Elsa Chkron (1) Céline Karabulut (1) Jérôme Lemoine (1) Noémie Trystram (1) Julien Vibert (1)

Department of Infectious Diseases

Pascal Crenn, MD, PhD (1,2,7) Benjamin Davido, MD, MS (1) Stéphanie Landowski, MD (1) Christian Perronne, MD, PhD (1,2) Véronique Perronne, MD (1) Pierre de Truchis, MD, MS (1)

Department of Infectious Diseases Interns

Marc Hobeika (1) Louis Jacob (1) Nicolas Kiavue (1) Aymeric Lanore (1) Aurélie Le Gal (1) Julia Nguyen Van Thang (1)

Department of Microbiology and Innovative Biomarkers Platform

Coralie Favier (1) Jean Louis Gaillard, MD, PhD (1,2,5) Elyanne Gault, MD, PhD (1,2,5) Jean-Louis Herrmann, PharmD, PhD (1,2,5) Christine Lawrence, PharmD (1) Virginie Lebidois, PharmD (1) Latifa Noussair, MD (1) Martin Rottman, MD, PhD (1,2,5) Anne-Laure Roux, PharmD, PhD (1,2,5) Sophie Tocqueville (1) Marie-Anne Welti, MD, PhD (1,2,5) And the nonmedical staff of the Department

Department of Laboratory Medicine and Pharmacology

Jean Claude Alvarez, MD, PhD (1,2,5) Mehdi Djebrani, PharmD (1) Pierre-Alexandre Emmanuelli (1) Firas Jabbour, PharmD (1) Lotfi Lahjomri, MD (1) Mathilde Parent, MD (1) And the nonmedical staff of the Department

Department of Radiology

Amine Ammar, MD (1) Najete Berradja, MD (1) Robert-Yves Carlier, MD, MS (1,2,7,14) Annaelle Chetrit, MD (1,2) Caroline Diffre, MD (1,2) Myriam Edjlali, MD, PhD (1,15) Zaki El Baz, MD (1,14) Adrien Felter, MD (1) Catherine Girardot, MD (1,13) Ahmed Mekki, MD, MS (1,2) Dominique Mompoint, MD (1) Dominique Safa, MD (1) Tristan Thiry, MD (1)

Department of Radiology Interns

Margot Armani (1) Olivier de Barry (1) Antoine Kirchner (1) Jeffery Zhou (1)

Department of Forensic Medicine

Geoffroy Lorin de La Grandmaison MD, PhD (1)

Department of Forensic Medicine Intern

Kevin Mahe (1)

Affiliations

1. Hôpital Raymond Poincaré, GHU APHP, Université Paris Saclay, Garches, France 2. Faculté Simone Veil Santé, Université Versailles Saint Quentin en Yvelines, Université Paris Saclay, Montigny-le-Bretonneux, France 3. Hôpital Foch, Suresnes, France 4. Centre Hospitalier Universitaire de Nantes, Nantes, France 5. Université de Versailles Saint-Quentin-en-Yvelines/INSERM, Laboratory of Infection & Inflammation–U-1173, Montigny-le-Bretonneux, France 6. Université de Versailles Saint-Quentin-en-Yvelines/INSERM, Centre d’Evaluation et de Traitement de la Douleur–U-987, Boulogne-Billancourt, France 7. Université de Versailles Saint-Quentin-en-Yvelines/INSERM, Handicap Neuromusculaire–U-1179, Montigny-le-Bretonneux, France 8. Centre d’Investigation Clinique, Garches, France 9. Commissariat à l’Energie Atomique, CEA Paris Saclay, Gif-sur-Yvette, France 10. Fondation Garches, Garches, France 11. Clinique Jouvenet, Ramsay Santé, Paris, France 12. Clinique de la Muette, Ramsay Santé, Paris, France 13. Polyclinique Mantaise, Mantes-La-Jolie, France 14. Centre Hospitalier Intercommunal Poissy/Saint-Germain, GHT Yvelines Nord, Poissy, France 15. IMA-BRAIN/INSERM–UMR-1266, DHU-Neurovasc, Centre Hospitalier Sainte-Anne, Paris, France

## Supplementary material

Supplementary Figure 1 Behavior of lymphocyte count and neutrophils over time from the admission (D0) according to oxygen level requirements
