# Supplementary figures and images for: Normalization of eosinophil count is predictive of oxygen weaning over the course of COVID-19 infection among hospitalized adults during the first wave of 2020 pandemic
Source: Front Immunol. 2024 May 24;15:1381059. doi: 10.3389/fimmu.2024.1381059 (PMC11157028; doi:10.3389/fimmu.2024.1381059)

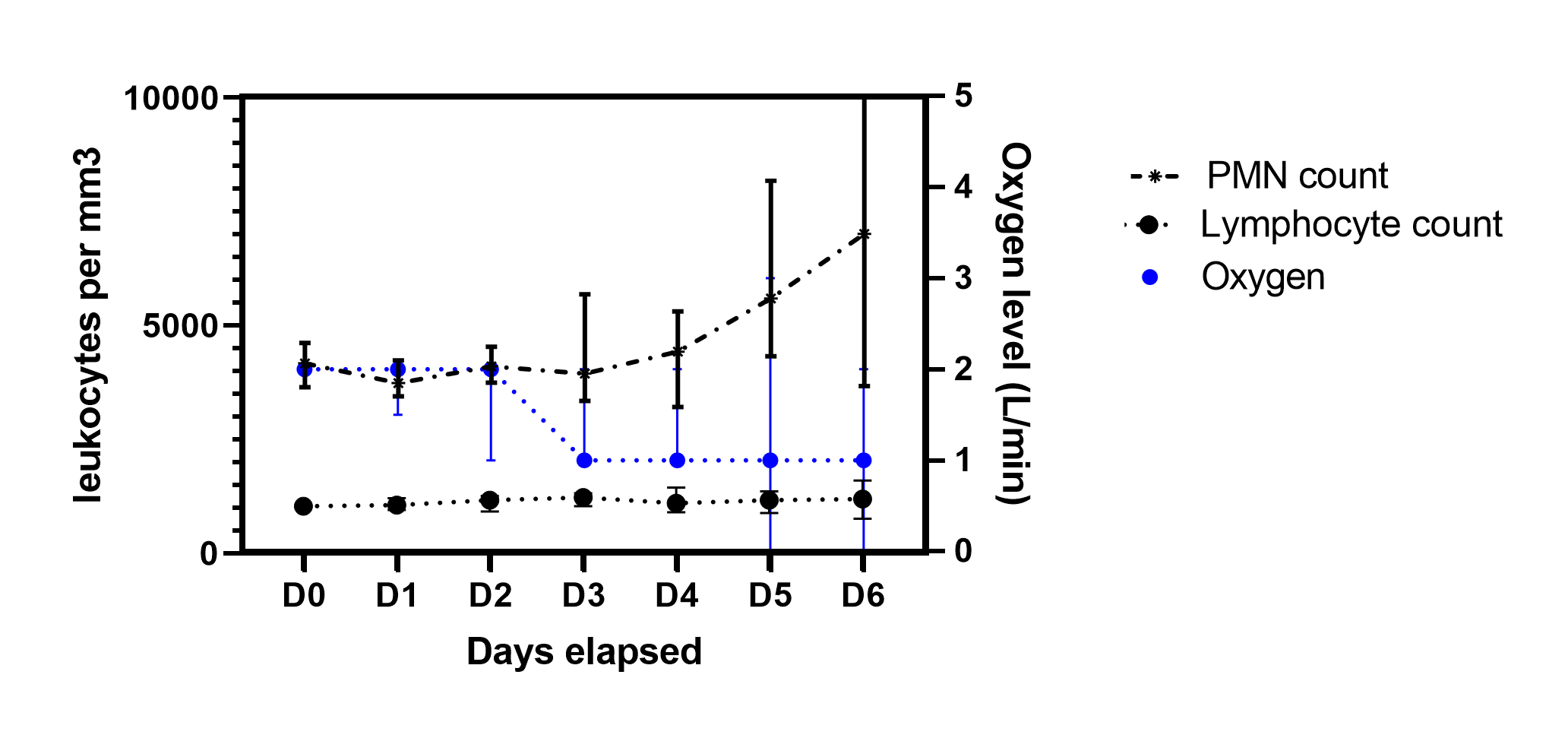

Supplement: Supplementary Figure 1 — Behavior of lymphocyte count and neutrophils over time from the admission (D0) according to oxygen level requirements [file Image_1.tif]
